# Coping Flexibility: Match Between Coping Strategy and Perceived Stressor Controllability Predicts Depressed Mood

**DOI:** 10.1007/s42761-024-00275-9

**Published:** 2024-10-05

**Authors:** Calissa J. Leslie-Miller, Jutta Joormann, Meghan E. Quinn

**Affiliations:** 1 Department of Psychology, William & Mary, Meghan Quinn, P.O. Box 8795, Williamsburg, VA 23187 USA; 2 Department of Psychology, Yale University, New Haven, CT USA; 3 Present Address: Department of Clinical Child Psychology, University of Kansas, Lawrence, KS USA

**Keywords:** Coping flexibility, Depression, Stress, Daily diary

## Abstract

Individual differences in coping responses can predict psychological distress, anxiety, and depression; therefore, it is vital to explore adaptive coping strategies. Recent research suggests that an individual’s ability to choose strategies based on the context may be more important than the ability to use any one strategy, an ability termed coping flexibility. For example, problem-focused coping is adaptive for situations of high control, while emotion-focused coping is adaptive for situations of low control. This conceptualization of coping flexibility, termed strategy-situation fit, consists of the match of strategy to situation. The purpose of the present study was to evaluate if daily fluctuations in strategy-situation fit for daily stressors would be associated with daily levels of depressed mood. A seven-day diary study in a sample of undergraduate students (*n* = 75) was completed. The results of generalized linear mixed models demonstrated that in situations of high stress and high control, more use of emotion-focused coping was related to higher levels of depressed mood. Additionally, in situations of high stress and low control, more use of emotion-focused coping was related to lower levels of depressed mood. These findings suggest that the match between emotion-focused coping and perceived stressor controllability can be a predictor of daily experiences of depressed mood when faced with high-level stressors.

Individuals regularly experience stressors of varying magnitudes and implications and must face the challenge of deciding how to respond to those stressors. Whereas stressors are particular events that elicit stress (Epel et al., 2018; Selye, 1956), stress has been identified as a transactional process determined by the appraisal of the stressor and an analysis of available resources (Lazarus & Folkman, 1984; Cohen et al., 2016). This process results in particular responses that are intended to manage the appraised demands of the stressor (Algorani & Gupta, 2024; Lazarus & Folkman, 1984). These responses, often referred to as coping strategies, can take many forms of thought or behavior. Thus, the experience of stress and which coping strategies are used may differ across individuals and within an individual from situation to situation. Coping strategy usage can predict psychological distress (Littleton et al., 2007), anxiety (Mahmoud et al., 2012), and depression (Joormann et al., 2006). Given the implications of how individuals cope with everyday stressors, it is vital to explore which coping strategies are most adaptive.

Coping strategies are often labeled as *problem-focused coping strategies*, aimed at altering the stressor itself, and *emotion-focused coping strategies*, intended to change how one feels about the situation (Lazarus & Folkman, 1984). A similar division is observed in other areas; for example, the motivational theory of lifespan development posits that individuals use primary control strategies (those aimed at changing their environment) and secondary control strategies (those focused on adapting oneself) to manage challenges (Heckhausen et al., 2010). Numerous studies have evaluated whether certain forms of emotion-focused or problem-focused coping strategies are most adaptive (Zeidner & Saklofske, 1996); however, an individual’s ability to flexibly choose strategies based on the context may be more important than the ability to use any one strategy (Bonanno & Burton, 2013). This ability to flexibly choose strategies is termed coping flexibility.

Previous research on coping flexibility has demonstrated its value; for example, individuals with higher levels of coping flexibility tend to report lower anxiety levels, fewer psychosomatic symptoms, fewer stress-related symptoms, and lower systolic blood pressure (Fresco et al., 2006; Watanabe et al., 2002). Although studies such as these support the importance of coping flexibility, they have not agreed on a single conceptualization of coping flexibility. In fact, five different conceptualizations are often used: broad repertoire, balanced profile, cross-situational variability, strategy-situation fit, and perceived ability (Cheng et al., 2014). There have been demonstrated benefits of greater coping flexibility using each of these different conceptualizations of coping flexibility, but studies report drastically different effect sizes when examining the link between coping flexibility and psychological adjustment (Cheng et al., 2014). Strategy-situation fit (i.e., the ability to match coping strategy choice to the demands of a stressor) tends to produce larger effect sizes than other conceptualizations of coping flexibility (Cheng et al., 2014), suggesting that it is more important to psychological adjustment.

Strategy-situation fit is largely based on person-situation interactionist theories that suggest a dynamic relationship between an individual’s appraisal of the demands of the stressor and how they chose to respond (Mischel & Shoda, 1995). This conceptual framework posits that selecting coping strategies tailored to the specific demands of a stressor is considered the most adaptive approach. The controllability of a stressor has been identified as a key situational characteristic that can distinguish adaptive and maladaptive matches between strategy choices and situations (Watanabe et al., 2002). Typically, problem-focused coping is a better fit for situations in which individuals have high levels of control because the source of the stress may be altered. In contrast, emotion-focused coping is a better fit for situations in which individuals have low levels of control because the emotional response to the stressor is all that can be manipulated (Lazarus, 1999).

Previous research on strategy-situation fit has demonstrated that the match between perceived stressor controllability and coping strategy usage (i.e., emotion-focused and problem-focused) is related to depression (Forsythe & Compas, 1987; Vitaliano et al., 1990; Zong et al., 2010). Vitaliano and colleagues (1990) found a negative relation between problem-focused coping and depression only when the stressor was perceived as controllable. They also reported a trend for a positive relationship between emotion-focused coping and depression for controllable stressors. Similarly, Forsythe and Compas (1987) found lower symptom scores in undergraduates when emotion-focused coping was used in situations of low control and when problem-focused coping was used in situations of high control. Additionally, Zong and colleagues (2010) found an association between lower levels of coping flexibility and high levels of depression, such that, compared to the non-depressed group, the depressed group used more problem-focused coping in situations of low control and less problem-focused coping in situations of high control. However, they found no significant group differences for emotion-focused coping.

Although studies such as these demonstrate a link between coping flexibility and depression, their design, involving data collection at a single time point, limits the interpretation of the data to between-subjects analyses. For example, participants in these studies report coping strategy usage in response to a single stressor. This means we cannot determine whether the reported coping strategy usage reflects a trait-like tendency to use particular strategies regardless of controllability of the stressor or whether the coping strategies may have been selected in response to the controllability of the stressor. Observing intraindividual variability in coping flexibility through daily diary methodology is one way to protect against the influence of stable, trait-like factors and instead observe how coping flexibility may relate to fluctuations in depressed mood. In other words, to better showcase the value of coping flexibility, studies could demonstrate that, within the same person, specific coping strategies may not be universally adaptive; rather, choosing certain strategies for certain situations may lead to better outcomes.

Previous research on emotion regulation flexibility provides important insight into the within-person, daily relationship between coping flexibility and depressed mood. Whereas *coping flexibility* focuses on managing various responses to stress, which includes but is not limited to emotions, *emotion regulation flexibility* focuses on managing emotions in a variety of contexts, not limited to stress (Carver, 2011; McRae & Gross, 2020). Despite this core difference, coping and emotion regulation flexibility share many features, including the core assertion that an individual’s ability to flexibly respond to the changing environment is adaptive (Aldao et al., 2015). Also mirroring coping flexibility, emotion regulation flexibility has been operationalized in a variety of different ways, including strategy-situation fit (Haines et al., 2016). For example, previous studies (Haines et al., 2016; Troy et al., 2013) have found that greater use of reappraisal (i.e., an emotion-focused strategy that involves changing how one thinks about a situation) in situations of low control and less use of reappraisal in situations of high control is associated with well-being. Similarly, Wenzel and colleagues (2020) found, through longitudinal ambulatory assessment, that the relationship between emotion regulation strategies and negative feelings was moderated by controllability, such that the effectiveness of interpersonal emotion regulation strategies depended on the controllability of the situation. These findings are consistent with research in motivational theory of lifespan development, demonstrating that secondary control strategies, such as goal adjustment and positive reappraisal, predict lower levels of depressive symptoms in situations of low control (Wrosch et al., 2000).

Applying a similar within-person design to coping flexibility research will allow us to better evaluate whether choosing a strategy that is a good fit for the situation may influence depressed mood. Unlike a between-person design that compares different individuals, a within-person design tracks variations within the same person and therefore is an ideal paradigm for testing the coping flexibility model. The present study aimed to demonstrate through 1 week of daily diaries that the match between coping strategy and the controllability of the situation predicts daily depressed mood within individuals. Notably, previous research on the goodness of fit conceptualization of coping flexibility has noted no effect for the relationship between coping flexibility and psychological symptomatology for *daily hassles*, that is, the repeated strains of everyday life (Forsythe & Compas, 1987); therefore, our hypotheses were specific to daily events rated high in perceived stress.

We hypothesized that at high levels of perceived stress, perceived stressor controllability would moderate the relation between emotion-focused coping and depressed mood. Specifically, we predicted that at lower levels of perceived controllability, more use of emotion-focused coping would be associated with lower levels of depressed mood. We also predicted that at higher levels of perceived controllability, more use of emotion-focused coping would be associated with higher levels of depressed mood. Additionally, at high levels of perceived stress, we hypothesized that perceived stressor controllability would moderate the relationship between problem-focused coping and depressed mood. In particular, we predicted that at higher levels of perceived controllability, more use of problem-focused coping would be associated with lower levels of depressed mood. We also predicted that at lower levels of perceived controllability, more use of problem-focused coping would be associated with higher levels of depressed mood.

## Method

### Participants

Seventy-eight college student participants were recruited through campus advertisements at a private university in the Northeastern United States for the parent study (Quinn & Joormann, 2020). Thus, the sample size was not selected for the present study; rather, participants in the present study were limited to 75 participants who completed at least one daily diary with a stressful event as part of their participation in the parent study (76 participants completed at least one daily diary and 75 completed at least one diary that reported a stressful event). Most participants were 18 years old (*M* = 18.51, *SD* = 0.60). The sample was 65.3% female and was racially and ethnically diverse, with 53.3% identifying as White or Caucasian, 41.3% as Asian or Asian American, 13.3% Latino or Hispanic, 9.3% Black, African American, or African, 1.3% American Indian, Native American, or Alaska Native, 5.3% Middle Eastern or Arab, and 2.7% as “Other.”

### Materials

#### Brief-COPE (Carver et al., 1989)

A 28-item self-report questionnaire was used to measure coping each day. In reference to the “most stressful” event of their day, participants indicate the degree to which they have been using coping strategies on a 4-point Likert scale ranging from 1 “I haven’t been doing this at all” to 4 “I’ve been doing this a lot.” This measure has demonstrated good internal consistency, test–retest reliability (Cooper et al., 2008), and concurrent validity (Su et al., 2015). The present study focused on strategies that could be grouped into emotion-focused coping (ω_within_ = .68; use of emotional support, positive reframing, humor, acceptance, and religion) or problem-focused coping (ω_within_ = .82; active coping, use of instrumental support, and planning). The emotion-focused and problem-focused coping composite scales have previously demonstrated good levels of internal consistency (Cooper et al., 2008; Kaiseler et al., 2012).

#### Perceived Stress Level

A single-item indicator was used to assess a participant’s perceived stress level of the greatest stressor of their day. Participants were asked how stressful the event was on a 5-point Likert scale ranging from 1 “not at all” to 5 “very stressful.”

#### Perceived Stressor Controllability

A single-item indicator was used to assess a participant’s perceived controllability of the greatest stressor of their day. Participants were asked how much they felt they had control over the outcome of the event on a 5-point Likert scale ranging from 1 “not at all” to 5 “very much.”

#### Depressed Mood

A single-item indicator was used to assess a participant’s daily experiences of depressed mood. Participants were asked to think about their experiences throughout the day and report the extent they felt depressed using a 5-point Likert scale ranging from 1 “not at all” to 5 “extremely.”

### Procedure

All study procedures were Institutional Review Board approved. Informed consent was received from all participants prior to completing any portion of the study, and participants were compensated monetarily for their time. Data for this study were collected for the parent study (Quinn & Joormann, 2020), in which participants completed either a laboratory stress induction or control task, along with a variety of surveys including a week of daily diaries. The present manuscript focuses exclusively on the daily diary portion of the parent study. The data presented in this manuscript are not presented elsewhere. Participants were emailed a daily diary at 8 pm each night for 7 days. Each day, participants were asked to think about the most stressful thing that happened to them that day. They described the event and rated its stressfulness, controllability, and resultant coping behaviors. On each day, participants also reported level of a variety of emotional outcomes (i.e., sad, anxious, alert) beyond the scope of this current manuscript, which focuses on depressed mood.

### Data Analysis

To evaluate whether strategy-situation fit for daily stressors would predict daily depressed mood, generalized linear mixed models (GLMM) were conducted. We used GLMMs due to the non-normal distribution of depressed mood. The probability distribution of the target was specified as gamma and identified as linearly related to the model effects via the log link function. Models fit by maximum likelihood estimation with a gamma distribution were estimated using the *lme4* package in R version 4.3.2. The models included random intercepts but not random slopes due to convergence limitations. All independent variables were person-centered, as we were interested in within-person associations. By using this design, day-to-day variation in appraisal of stressor controllability could be analyzed as a moderator of the association between day-to-day variability in coping strategy usage (i.e., emotion-focused or problem-focused) and daily depressed mood. Analyses were run separately for emotion-focused and problem-focused coping. In the first model, predictors of depressed mood were emotion-focused coping, controllability, stress level, emotion-focused coping*controllability, emotion-focused coping*stress level, controllability*stress level, and emotion-focused coping*stress level*controllability. In the second model, predictors of depressed mood were problem-focused coping, controllability, stress level, problem-focused coping*controllability, problem-focused coping*stress level, controllability*stress level, and problem-focused coping*stress level*controllability. In each model, the key predictor was the three-way interaction between coping, stress level, and controllability.

Significant interactions were plotted and probed using the *sjplot* and *interactions* packages in R. We examined the relations between coping and depressed mood at mean levels of controllability as well as one and two *SD*s above and below the mean level of controllability. These relations were examined separately at mean and one *SD* above and below the mean level of stress. Johnson-Neyman regions of significance were also computed to determine the levels of controllability at which coping and depressed mood were significantly related. These analyses were not preregistered.

## Results

Of the 532 potential surveys from the 76 participants who participated in the daily diary portion of the study, 97 surveys were incomplete and 26 surveys were excluded because they reported no stress (i.e., stress level was rated “not at all” and associated events included descriptions such as “seeing a friend” and “there was nothing stressful in my day today”). This resulted in data for 75 participants (409 daily diaries), which were included in analyses. The proportion of level 1, within-person, variance of depressed mood was .68. Prior to person-centering independent variables, scores were as follows: emotion-focused coping (*M* = 1.70, *SD* = 0.50), problem-focused coping (*M* = 1.97, *SD* = 0.79), controllability (*M* = 2.50, *SD* = 1.16), and stress level (*M* = 3.21, *SD* = 0.95). After person-centering independent variables, emotion-focused coping scores ranged from − 1.25 to 2.08 (*M* = .00, *SD* = 0.35), problem-focused coping scores ranged from − 1.42 to 2.08 (*M* = .00, *SD* = 0.58), controllability ratings ranged from − 2.67 to 3.14 (*M* = .00, *SD* = 1.00), and stress level (*M* = .00, *SD* = 0.77). Depressed mood was not person-centered, so the range remained 1 to 5 (*M* = 1.39, *SD* = 0.70).

### Primary Analyses

**Fig. 1:**
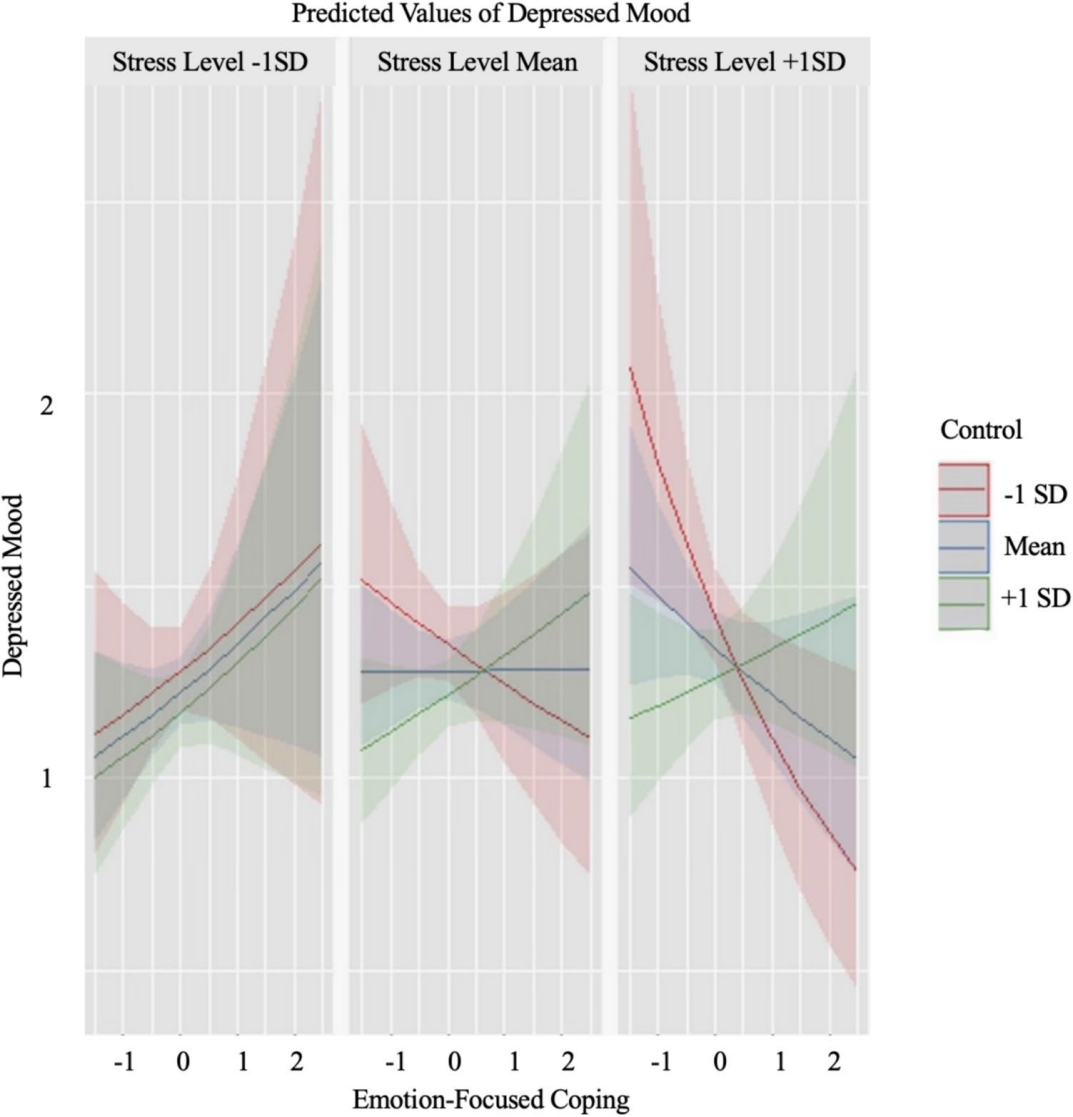
The interaction between emotion-focused coping and perceived stressor controllability (+ / − 1*SD*) at varying levels of stress (409 observations)

**Fig. 2:**
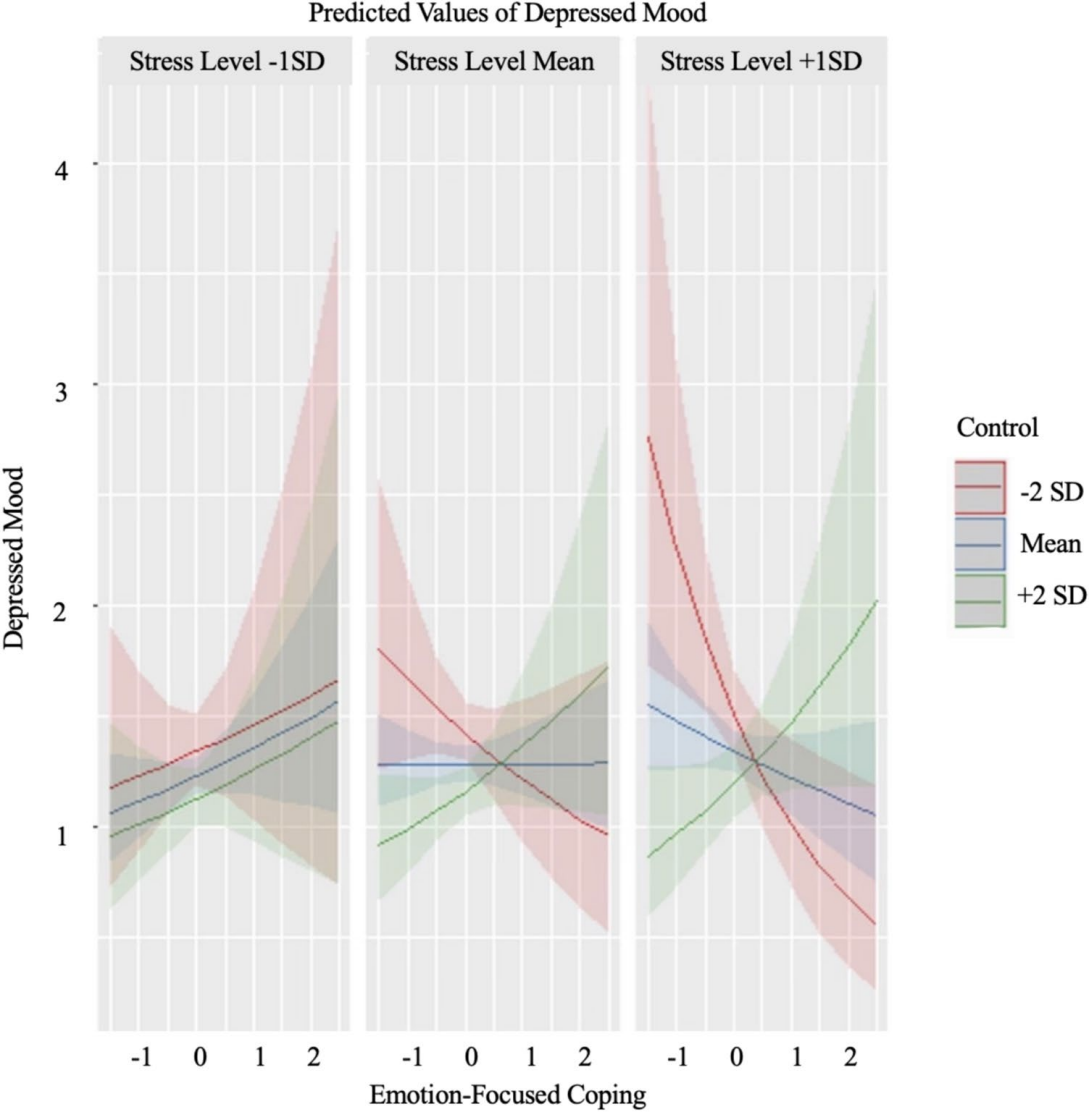
The interaction between emotion-focused coping and perceived stressor controllability (+ / − 2*SD*) at varying levels of stress (409 observations)

Results from GLMMs are presented in Table 1. As hypothesized, there was a significant interaction between emotion-focused coping, perceived stressor controllability, and stress level. To follow up on the significant 3-way interaction, at the mean level of stress and one standard deviation above and below the mean level of stress, we assessed the association between emotion-focused coping and depressed mood throughout the range of controllability ratings (i.e., mean controllability ratings as well as one (see Fig. 1) and two (see Fig. 2) standard deviations above and below mean controllability). When the event was perceived as more stressful than average (+ 1 *SD*), the relation between emotion-focused coping and depressed mood was significant at two standard deviations above the mean of controllability (*ß* = .21, *p* = .03), but was not significant at one standard deviation above the mean of controllability (*ß* = .06, *p* = .36) nor mean levels of controllability (*ß* =  − .10, *p* = .10). Further, when the event was perceived as more stressful than average (+ 1 *SD*), the relation between emotion-focused coping and depressed mood was significant at one standard deviation below the mean of controllability (*ß* =  − .25, *p* = .01) and two standard deviations below the mean of controllability (*ß* =  − .40, *p* < .01), such that more use of emotion-focused coping was related to lower levels of depressed mood. Johnson-Neyman intervals indicate that, at high levels of stress, the relation between emotion-focused coping and depressed mood was significant when controllability ratings were outside of the interval [− .19, 1.67], which is within the range of observed values [− 2.67, 3.14].

**Table 1:** Results from GLMMs of daily associations between coping strategy usage and depressed mood moderated by perceived stressor controllability and stress level (409 observations)

Variable	Estimate	*SE*	*t*	*p*	*95% CI*	
					Lower	Upper
Emotion-focused						
Intercept	.25	.04	5.66	** < .001**	.17	.33
Coping	.00	.04	.03	.980	− .08	.08
Control	− .05	.02	− 3.37	** < .001**	− .09	− .01
Stress	.06	.02	2.95	**.003**	.02	.10
Coping*Control	.08	.04	1.84	.066	.00	.16
Coping*Stress	− .13	.06	− 2.19	**.029**	− .25	− .01
Control*Stress	− .01	.02	− .47	.639	− .05	.03
Coping*Control*Stress	.10	.04	2.26	**.024**	.02	.18
Problem-focused						
Intercept	.25	.04	5.56	** < .001**	.17	.33
Coping	− .03	.03	− 1.13	.257	− .09	.03
Control	− .05	.02	− 2.84	**.005**	− .09	− .01
Stress	.06	.02	2.77	**.006**	.02	.10
Coping*Control	.02	.03	.91	.364	− .04	.08
Coping*Stress	− .03	.04	− .75	.456	− .11	.05
Control*Stress	− .01	.02	− .44	.663	− .05	.03
Coping*Control*Stress	.04	.02	1.75	.080	.00	.08

At mean levels of stress, the relation between emotion-focused coping and depressed mood was not significant at one (*ß* = .08, *p* = .14) or two (*ß* = .16, *p* = .07) standard deviations above the mean of controllability, nor at mean levels of controllability (*ß* = .00, *p* = .99), nor at one (*ß* =  − .08, *p* = .26) or two (*ß* =  − 16, *p* = .14) standard deviations below the mean of controllability.

When the event was perceived as less stressful than average (− 1 *SD*), the relation between emotion-focused coping and depressed mood was not significant at one (*ß* = .10, *p* = .20) or two (*ß* = .11, *p* = .38) standard deviations above the mean level of controllability, nor at mean levels of controllability (*ß* = .10, *p* = .14), nor at one (*ß* = .09, *p* = .33) or two (*ß* = .09, *p* = .54) standard deviations below the mean of controllability.

Contrary to our hypothesis, we did not find evidence for a significant interaction among stress level, stressor controllability, and problem-focused coping predicting depressed mood.

### Exploratory Analyses

Exploratory analyses were conducted to follow up on the significant three-way interaction among emotion-focused coping, controllability, and stress level predicting depressed mood. First, we examined whether any individual strategy may have been driving this effect. We ran the same model described previously, but emotion-focused coping was replaced with each individual strategy (i.e., emotional support, positive reframing, humor, acceptance, religion) in five separate models. We did not observe significant three-way interactions among coping, controllability, and stress level with emotional support (*b* = .04, *p* = .185), humor (*b* = .04, *p* = .281), or religion (*b* = .04, *p* = .422). A significant three-way interaction among coping, controllability, and stress level was observed with positive reframing (*b* = .05, *p* = .039) and acceptance (*b* = .06, *p* = .018). Probing each of these significant interactions revealed patterns similar to what was observed with the emotion-focused coping composite. Johnson-Neyman intervals indicate that, at high levels of stress, the relation between positive reframing and depressed mood was significant when controllability ratings were outside of the interval [− .65, 1.76], which is within the range of observed values [− 2.67, 3.14]. Johnson-Neyman intervals indicate that, at high levels of stress, the relation between acceptance and depressed mood was significant when controllability ratings were outside of the interval [− .18, 2.38], which is within the range of observed values [− 2.67, 3.14].

The second exploratory analysis was to examine whether the interaction among emotion-focused coping, controllability, and stress level would predict depressed mood the following day. We ran the same model described previously but to test lagged effects, the outcome was depressed mood on the subsequent day, and depressed mood on the current day was included as a predictor. A significant three-way interaction among emotion-focused coping, controllability, and stress level was observed when predicting depressed mood the following day (*b* = .12, *p* = .007). Probing the significant interaction revealed patterns similar to what was observed with the original model. Johnson-Neyman intervals indicate that, at high levels of stress, the relation between positive reframing and depressed mood was significant when controllability ratings were outside of the interval [− .82, 1.65], which is within the range of observed values [− 2.67, 3.14].

## Discussion

The purpose of the present study was to demonstrate that coping flexibility, conceptualized as the ability to match coping strategies to the perceived controllability of a stressor, was associated with daily levels of depressed mood. Specifically, we were interested in observing that within the same person, specific coping strategies may not be universally adaptive; rather, choosing strategies that are a better match for the situation may predict lower depressed mood. Although our design was correlational, it provides a better test of the idea that strategy-situation fit is important, because it mitigates the influence of stable trait-like factors and shows that the effectiveness of coping strategies is context-dependent. This underscores the potential importance of coping flexibility in managing daily stressors to reduce depressed mood.

At higher levels of stress, the match between perceived stressor controllability and emotion-focused coping predicted depressed mood, such that when perceived stressor controllability was higher, greater use of emotion-focused coping was associated with higher ratings of depressed mood. This finding is consistent with cross-sectional research demonstrating a trend for a positive relationship between emotion-focused coping and depression for controllable stressors (Heckhausen et al., 2010; Vitaliano et al., 1990). Given the correlational nature of our study, results such as these can be interpreted in several ways. One possibility is that use of emotion-focused coping may be harmful when the situation is appraised as highly controllable (Herman & Tetrick, 2009). This is consistent with prior studies demonstrating that poor coping flexibility contributes to psychopathology vulnerability (Cheng et al., 2012). It is also possible that depressed mood contributed to the tendency to use emotion-focused coping for stressors perceived as more controllable. This interpretation is consistent with previous demonstrations that individuals with low self-esteem and depressed mood are more likely to select emotion-focused coping strategies (Li et al., 2023; Seiffge-Krenke, 1995)**.**

Additionally, at higher levels of stress and lower levels of control, emotion-focused coping was significantly related to depressed mood, such that greater use of emotion-focused coping was related to lower ratings of depressed mood. These results are consistent with Forsythe and Compas (1987), who demonstrated that emotion-focused coping is a better fit for situations in which individuals have lower levels of control. This suggests that emotion-focused coping is adaptive for stressors perceived as lower in controllability.

It is also worth noting that we found a significant negative relationship between perceived control and depressed mood. Although this cannot be clearly interpreted given the observed significant interactions that include perceived control, this is not surprising given the extensive research demonstrating that individuals who perceive a lack of control are more likely to experience depressed mood (Brown & Siegel, 1988) and those with depression may have a more pessimistic view of their controllability (Anderson et al., 2023; Moore & Fresco, 2012).

To follow up on our significant three-way interaction among stress level, controllability, and emotion-focused coping, we conducted exploratory analyses to see if any one strategy was driving this effect. Exploratory analyses conducted on each of the five strategies revealed significant three-way interactions among coping, controllability, and stress level when positive reframing and acceptance were the coping strategies entered in the model, but not when emotional support, humor, or religion were examined. The results for positive reframing and acceptance mirrored the patterns observed when the emotion-focused composite was entered in the model, suggesting that these two strategies may have been driving the overall effect. These results indicate that use of positive reframing and acceptance may play a role in mitigating the impact of stress on mood when individuals perceive their stressor as uncontrollable. While this study supports common recommendations for the use of these strategies (Aldao et al., 2010), it also introduces a caveat. Given that more use of these strategies in controllable situations was associated with higher depressed mood, they may not always be the optimal choice in every context.

In additional exploratory analyses, we found a significant three-way interaction among emotion-focused coping, controllability, and stress level when predicting depressed mood on the subsequent day, while controlling for depressed mood on the current day. The patterns observed were consistent with the original model, reinforcing that the effectiveness of coping strategies is context-dependent. Although these data are correlational, this temporal prediction supports the possibility that coping flexibility is influencing depressed mood, rather than depressed mood influencing coping flexibility. These results are also important because they suggest that the effects of coping flexibility may have lasting impacts on mood.

We did not find evidence for a link between strategy-situation fit and depressed mood when problem-focused coping was examined. These results are inconsistent with a previously reported negative relation between problem-focused coping and depression in situations of high control (Vitaliano et al., 1990); though, Vitaliano and colleagues (1990) did not observe a significant relation between problem-focused coping and depression at low levels of control. There are many possible explanations for these inconsistencies. One possibility may stem from the use of problem-focused coping measures. This is an umbrella term consisting of many different strategies, and results may differ depending on which strategies were captured by the coping measure used in each study. It is also possible the discrepancies between our findings and others are due to our use of a within-person design. Within- and between-person designs can produce very different, even opposite results (Curran & Bauer, 2011; Hamaker & Wichers, 2017).

Indeed, the use of a within-person design in the present study is an important extension of prior research. In fact, a within-person design may be most appropriate when stress is viewed as a transactional process in which coping choices differ depending on the attributes of the stressor encountered and an individual’s appraisal of the stressor (Lazarus & Folkman, 1984). This means that coping choices are not necessarily stable within individuals. Because coping strategy selection can vary across situations within individuals, the likelihood of experiencing depressed mood following a stressor may be partially dependent on the coping choices made in response to that particular stressor. Between-subjects designs cannot account for such intraindividual variability and therefore cannot fully interrogate the link between coping flexibility and depressed mood.

Our study includes limitations such as the possibility of inflated type I and type II errors because the models used in this study constrained individuals to share a common slope (Barr et al., 2013). Allowing the slope to vary was not feasible with the data structure and remains a limitation. Additionally, we decided to use single-item indicators for perceived stressor controllability and depressed mood to avoid respondent burden (Fisher et al., 2015). Daily diary designs often use single item measures because of the number of times people complete the same measure and, in fact, benefit from doing so (Fu, 2005). However, future research should seek to include scales that encompass greater depression symptomatology.

We evaluated perceived stressor controllability which can be greatly influenced by personal experiences, cognitive biases, psychological states, and external feedback (Folkman, 2013). In addition to perceived controllability shaping coping strategy choices, coping strategies can influence perceptions of controllability (Cheng et al., 2014). Because perceptions of controllability and coping strategies were recorded at the same time after the stressor, it is possible that perceptions of controllability influence coping strategy choices and vice versa. Further, consistent with the motivational theory of lifespan development, the accuracy of perceived controllability increases with age (Raab et al., 2022); therefore, these relationships may look different in samples of different ages (e.g., children or older adults). Future research should aim to disentangle the time ordering of observations to better understand the dynamic interplay between coping strategies and perceived stressor controllability within varying age groups.

Although the sample was racially diverse, our participants were all recruited through a college campus and of college-age; therefore, our results may not generalize to non-college student samples. Additionally, our small sample size may have constrained the broader application of our results. Caution is warranted in interpreting both our significant findings related to emotion-focused coping and our null findings concerning problem-focused coping. Relatedly, we did not conduct a power analysis for this study, which is a common limitation in many daily diary and other intensive longitudinal designs. Due to the novelty of our study design, we did not have existing data to estimate all of the parameters needed to determine the appropriate sample size for achieving desired statistical power. Future research should seek to rectify these limitations through increased sample size, longer data periods, and more diverse samples. Additionally, since perceived stressor controllability is just one factor of many potentially relevant factors, future research should explore additional contextual factors that might influence the context in which strategies are most adaptive. It is also important to note that our study relies on correlational data, and we are unable to determine whether depressed mood resulted from the stressor and coping response or vice versa. Although our exploratory lagged analyses support the possibility that coping impacts depressed mood, future research designed to test temporal or causal relations should attempt to replicate these findings.

Overall, the findings of the present study highlight the importance of the fit between contextual elements of a stressor and the coping strategies that are implemented. In particular, the match between emotion-focused coping and perceived stressor controllability can be a predictor of daily experiences of depressed mood. Notably, this relationship was observed in a within-person design, which mitigates the influence of stable trait-like factors, offering a clearer picture of how coping strategies function. This approach shows that the association between coping strategies and depressed mood is context-dependent, varying according to the specific circumstances and perceptions of the individual on any given day.

## Additional Information

### Data Availability

Data and analytic code are available from the corresponding author upon request.
